# A Novel Nomogram for Predicting the Survival of Patients with Invasive Upper Tract Urothelial Carcinoma

**DOI:** 10.7150/jca.50419

**Published:** 2021-01-01

**Authors:** Zaishang Li, Xueying Li, Yonghong Li, Ying Liu, Peng Du, Zenqing Liu, Kefeng Xiao

**Affiliations:** 1 Department of Urology, Shenzhen People's Hospital, The Second Clinic Medical College of Jinan University 518060, Shenzhen, Guangdong, P. R. China.; 2 Department of Urology, First Affiliated Hospital of Southern University of Science and Technology, 518060, Shenzhen, Guangdong, P. R. China.; 3 Department of Urology, Shenzhen Engineering and Technology Center of minimally Invasive Urology, Shenzhen People's Hospital, 518060, Shenzhen, Guangdong, P. R. China.; 4 Department of Oncology, The Seventh Affiliated Hospital Sun Yat-sen University, 518107, Shenzhen, Guangdong, P. R. China.; 5 Department of Urology, Sun Yat-sen University Cancer Center, 510060, Guangzhou, Guangdong, P. R. China.; 6 State Key Laboratory of Oncology in South China, 510060, Guangzhou, Guangdong, P. R. China.; 7 Collaborative Innovation Center of Cancer Medicine, 510060, Guangzhou, Guangdong, P. R. China.; 8 Gynecology Department, Long-gang District Maternal and Child Healthcare Hospital, 518172, Shenzhen, Guangdong, P. R. China.

**Keywords:** urothelial carcinoma, upper urinary tract, prognosis, radical nephroureterectomy, mortality

## Abstract

**Purpose:** Available tools for the prediction of the prognosis of patients with upper tract urothelial carcinoma (UTUC) are unified. We determined whether a novel nomogram is effective in estimating the survival of patients with invasive UTUC.

**Methods:** From January 2004 to December 2015, 4796 invasive UTUC patients in the Surveillance, Epidemiology and End Results database underwent radical nephroureterectomy (RNU) for invasive UTUC. The medical records of the patients were randomly (7:3) divided into the training and validation cohorts. The independent factors included in the nomogram were selected by multivariate analyses. The nomogram was developed based on the training cohort. Bootstrap validation was applied to validate the nomogram, whereas external validation was performed using the validation cohort. The accuracy and discrimination of the nomogram were assessed using concordance indices (C-indices) and calibration curves.

**Results:** The multivariate Cox regression model identified that age, tumor stage, node stage, metastasis stage and grade were associated with survival. In the training set, the nomogram, which included the above factors, exhibited discrimination power superior to that of the 8th American Joint Committee on Cancer (AJCC) TNM classification (Harrell's C-index, 0.74 vs. 0.71; *P* < 0.001). The nomogram showed better probability of survival agreement with the C-index than the AJCC-TNM staging system in the bootstrap validation** (**0.74 vs. 0.70; *P* < 0.001**)** and validation set (Harrell's C-index, 0.77 vs. 0.73; *P* < 0.001). The validation revealed that this nomogram exhibited excellent discrimination and calibration capacities.

**Conclusion:** An accurate novel nomogram that is superior to the current AJCC-TNM staging system was established for the prediction of CSS after RNU for invasive UTUC.

## Introduction

Urothelial carcinomas (UCs) are the fourth most common tumors. However, upper tract urothelial carcinomas (UTUCs) are uncommon and account for only 5-10% of all UCs, and 60% of UTUCs are invasive at diagnosis 1, 2. Radical nephroureterectomy (RNU) is the standard surgical treatment for patients with invasive UTUC 3, 4.

Due to individual differences, patients' prognosis significantly varies. Patients' prognosis can be predicted by clinicopathological factors, such as the T stage, N stage and grade 5-7. However, the prognosis of patients cannot equally and accurately be reflected by a single factor. The European Association of Urology Guidelines on Upper Urinary Tract Urothelial Carcinoma refers to several nomograms 8. Different nomograms incorporating various variables have been developed and are even based on the same French collaborative national database 9-11. Several nomograms include blood parameters, radiographic parameters or immunohistochemical markers 12, 13. Other nomograms include ≥6 clinicopathological features 10, 14. Therefore, a unified standard for nomograms is lacking.

The purpose of this study is to explore whether common factors could be used to develop a concise prediction model. To address the void in the ability to predict the prognosis of patients with UTUC, we developed a new model for the prediction of cancer-specific mortality (CSS) after RNU.

## Materials and methods

### Study population

Patients diagnosed with UTUC (codes ICD-O-2 C65.9 and C66.9) between 2004 and 2015 with available TNM stage classification data were identified from 18 Surveillance, Epidemiology and End Results (SEER) registries. Data from Alaska; Atlanta; California, except for San Francisco (SF)/San Jose/Monterey (SJM)/Los Angeles (LA); Connecticut; Detroit; Greater Georgia; Hawaii; Kentucky; Los Angeles; Rural Georgia; Louisiana; Iowa; New Jersey; New Mexico; San Francisco; San Jose; Seattle; and Utah were obtained. An independent pathological committee reviewed all histopathology findings 15.

Patients with detailed clinicopathological information who underwent RNU for invasive UTUC were considered. The detailed eligibility criteria were as follows: 1) histologically confirmed UTUC, 2) RNU for a primary lesion, 3) definite pathological staging, 4) detailed medical records, and 5) follow-up ≥1 month. The data with the highest level of histopathology or data concerning the primary tumor were considered.

### Methods

The cause of death was defined according to the SEER mortality cause assignment. The outcome of interest was CSS. The medical records of the patients were randomly (7:3) divided into the training (n=3360) and validation (n=1436) cohorts. The Kaplan-Meier method was used to determine CSS.

The log-rank test was used to compare the CSS rates among the different groups. A logistical Cox regression model was used to select the most useful prognostic markers of CSS. For the AJCC-pathological prognostic group classification, including the tumor stage, node stage, and metastasis stage, only the latter factors were included in the multivariate analysis. Accordingly, a nomogram for the prediction of the individual probability of CSS was developed based on the training cohort. Bootstrap validation (resampling with 1000 iterations) was applied to validate the nomogram, whereas external validation was performed using the validation cohort.

For the model validation, we assessed its discrimination and calibration. Discrimination was measured using the c-index. The statistical analyses were performed, and a two-sided P<0.05 was considered significant. The models, statistical analysis, and figures were prepared using SAS 9.4 software version (Cary, NC) and R 3.5.1 (http://www.cran.r-project.org).

## Results

In total, 4796 patients who underwent RNU for invasive UTUC were randomly (7:3) divided into the training (n=3360) and validation (n=1436) cohorts. In the training cohort, the median age at diagnosis was 73 years (range, 30-96 years). The median age at diagnosis in the validation cohort was 73 years (range, 38-101 years). **Table 1** shows the detailed clinicopathological characteristics of the cohorts.

**Figure 1:**
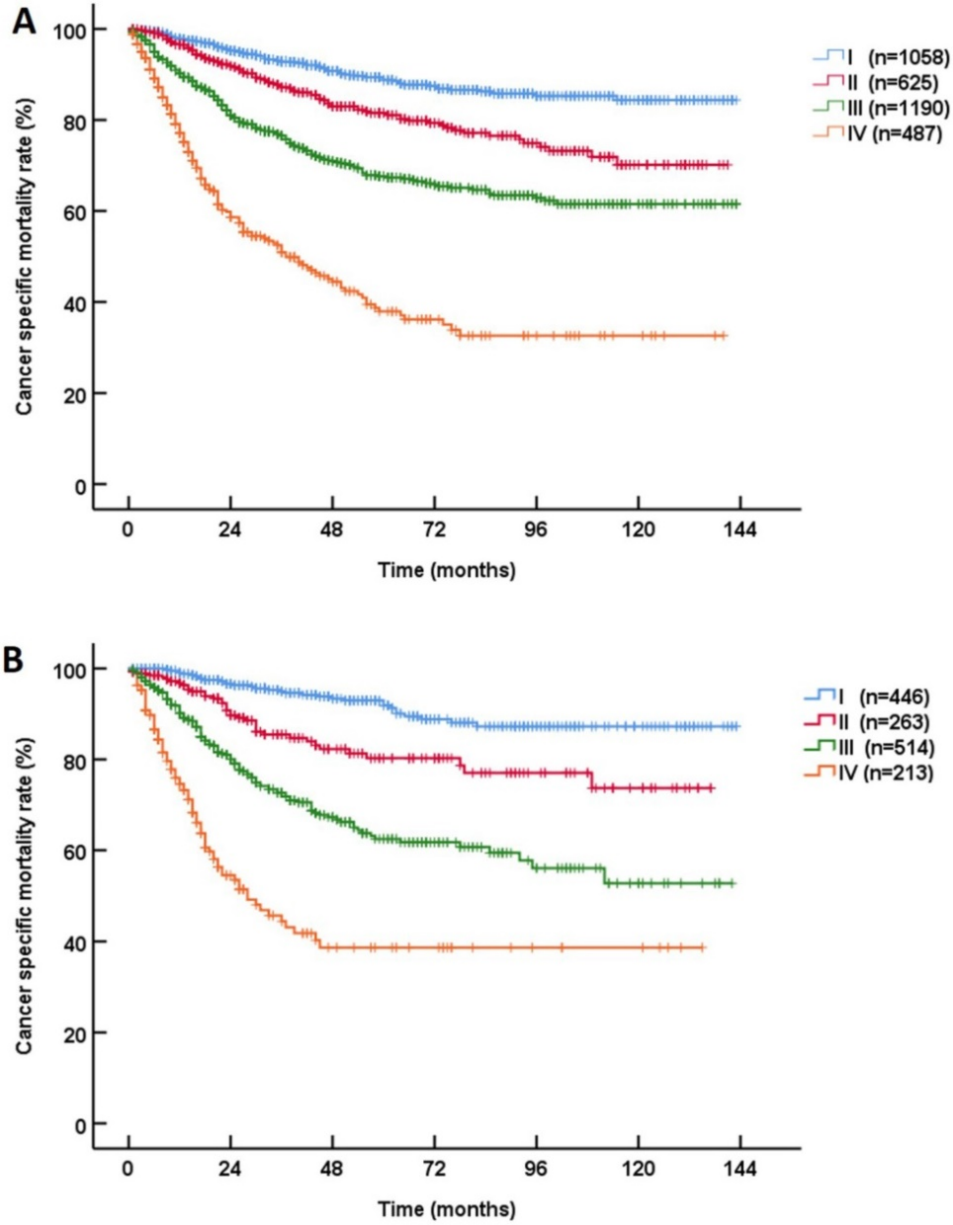
Kaplan‑Meier Cancer-specific survival (CSS) curves of patients with invasive upper tract urothelial carcinoma in different AJCC-pathological prognostic group classifications. **A:** training cohort; **B:** validation cohort.

At 5 years after RNU, 19.8% (946/4769) of the UTUC patients died. According to the most recent pathological prognostic group classification, the 3-year CSS rates of patients with stage I, II, III and IV disease were 93.5%, 86.7%, 75.4% and 49.0%, and the 5-year CSS rates of patients with stage I, II, III and IV disease were 89.7%, 81.1%, 66.1% and 38.1%, respectively (*P*<0.001,** Figure 1**). The stratified Kaplan-Meier plots shown in **Table 2** illustrate the effects of various factors on the actual CSS rates. In the training cohort, the univariable analyses revealed that age, gender, the tumor stage, the node stage, the metastasis stage and the grade were statistically significantly associated with CSS (all *P*<0.001).

**Figure 2:**
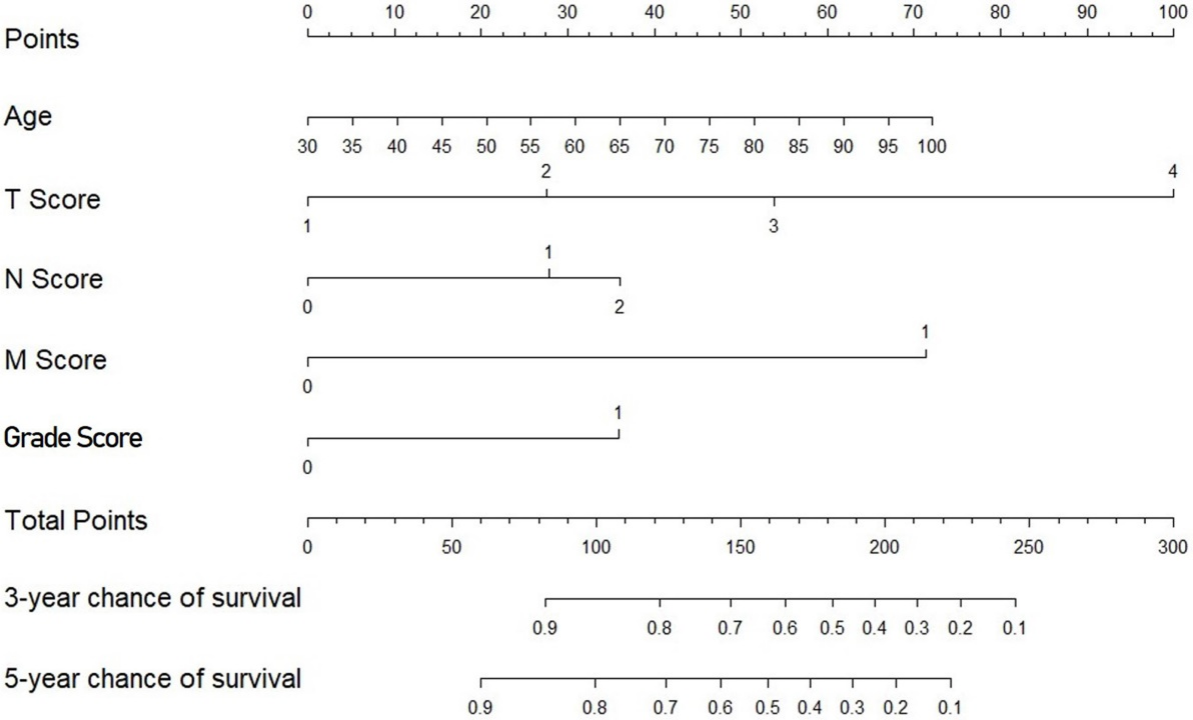
Nomogram predicting survival in patients with invasive upper tract urothelial carcinoma.

The multivariate Cox regression model identified age, the tumor stage, the node stage, the metastasis stage and the grade as covariates associated with survival (all *P*<0.001) (**Table 3**). The nomogram subsequently generated based on this multivariate analysis is shown in **Figure 2.**

**Figure 3:**
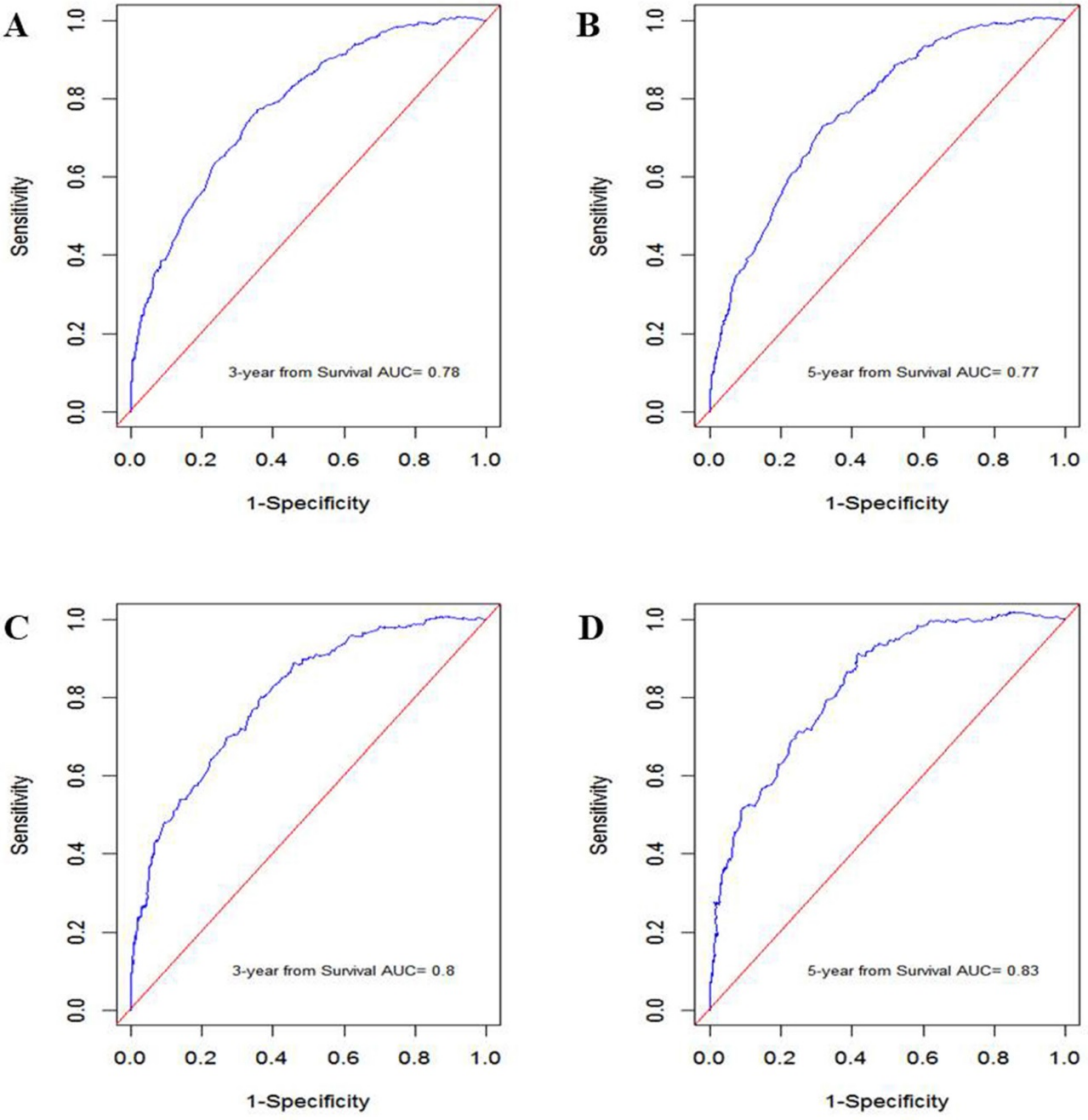
ROC curve generated by the nomogram. **A:** 3-year CSS in the training cohort. **B:** 5-year CSS in the training cohort. **C:** 3-year CSS in the validation cohort. **D:** 5-year CSS in the validation cohort.

**Figure 4:**
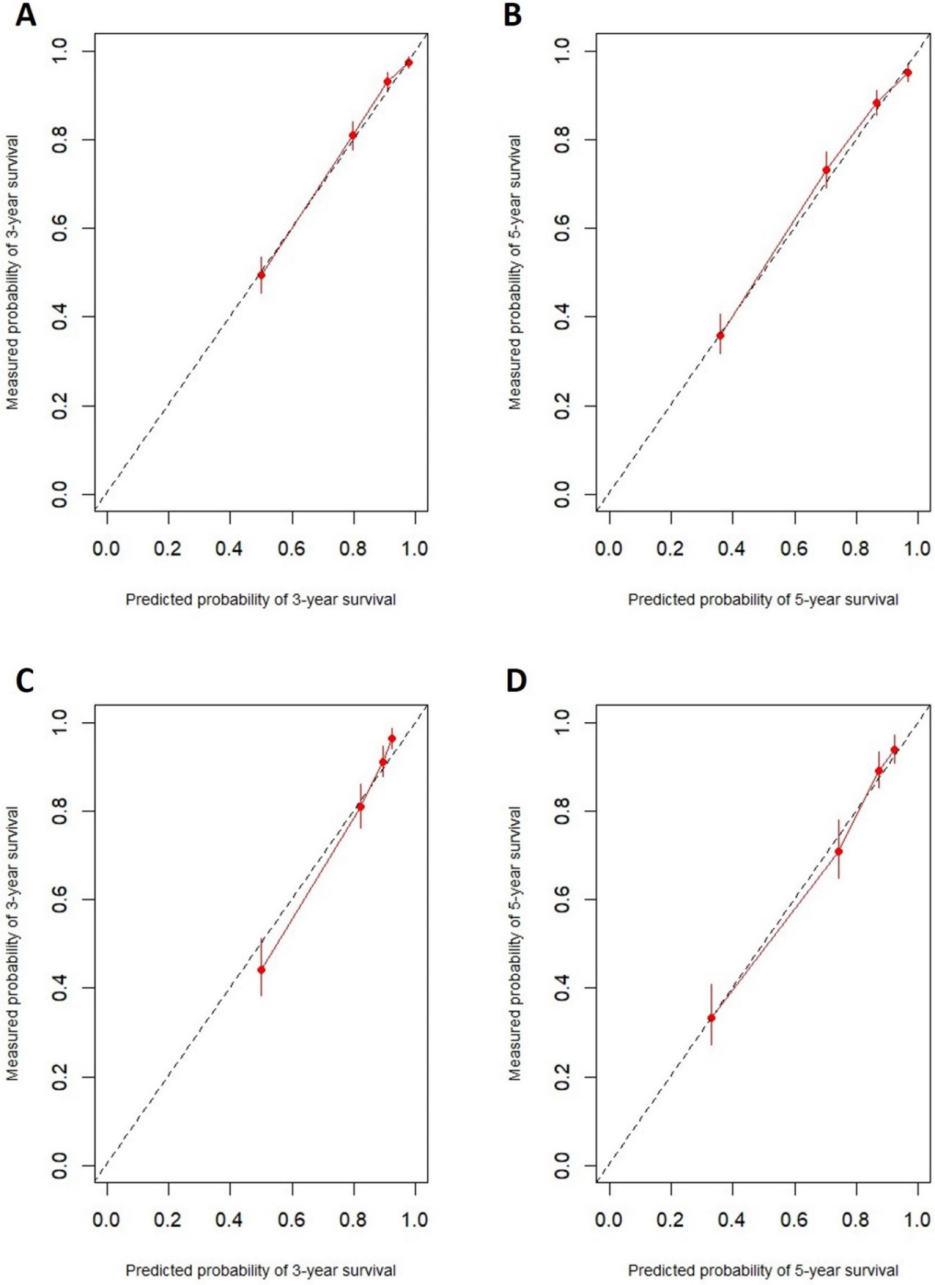
The prognostic accuracy of the nomogram in patients with invasive upper tract urothelial carcinoma. **A:** 3-year CSS in the training cohort. **B:** 5-year CSS in the training cohort. **C:** 3-year CSS in the validation cohort. **D:** 5-year CSS in the validation cohort.

**Figure 5:**
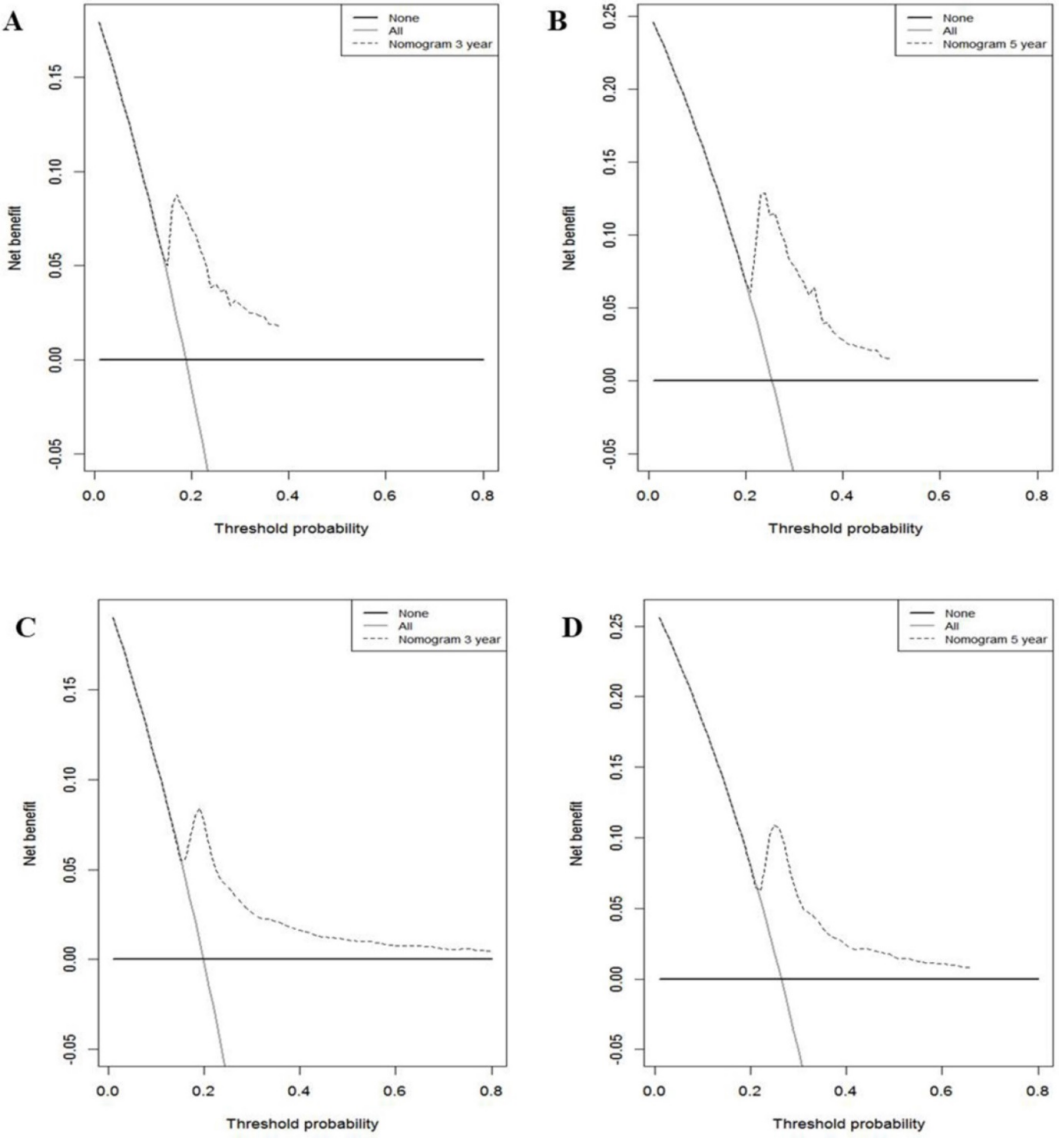
Decision curve analysis used to assess the clinical usefulness of the nomogram. **A:** 3-year CSS in the training cohort. **B:** 5-year CSS in the training cohort. **C:** 3-year CSS in the validation cohort. **D:** 5-year CSS in the validation cohort.

In the training cohort, the nomogram exhibited discrimination power superior to that of the 8th AJCC TNM classification (Harrell's concordance index [C-index], 0.74 vs. 0.71; *P*<0.001). The bootstrap-corrected C-index of the nomogram was 0.74, which was inferior to that of the 8th AJCC TNM staging system (C-index, 0.70; *P*<0.001). The receiver operating curve is illustrated in** Figure 3.** The calibration plots and decision curve analysis suggested that the accuracy in predicting CSS was good as shown in **Figure 4, Figure 5** and **Table 4.**

## Discussion

CSS after RNU for UTUC may be highly variable 2. Clinicopathological factors, such as the T stage, N stage and G stage, represent established predictors of prognosis 5, 14. Few nomograms incorporate various variables as predictors of prognosis 9-11. However, a unified standard for nomograms is lacking.

Using the SEER database, we developed a concise nomogram for the prediction of CSS in patients with UTUC and verified its clinical application value. The nomogram, which includes only 5 of the most common factors, is concise and has high applicability. The results were obtained in the training cohort and tested in the validation cohort. This model represents an individualized prognostic tool for patients treated with RNU based on the largest UTUC data set.

Invasive UTUC has a very poor prognosis 1, 2. In this study, RNU afforded a 3-year and 5-year CSS rate of 69% and 73%, respectively 6. Several single-center series involving >200 patients published to date have demonstrated 5-year CSS rates ranging from 61%-76% after RNU 16, 17. According to the currently accepted definition, the AJCC TNM staging system is considered among the most important prediction tools for UTUC. In our study, the 5-year CSS rates of the patients with stage I, II, III and IV disease were 87.3%, 79.3%, 64.9% and 32.8%, respectively. Although this finding represents a monumental step toward the goal of precision medicine, this work was published only very recently.

The main prognostic factors include preoperative and postoperative factors. Age, the tumor stage, the node stage, the metastasis stage and the grade, which are the most common factors, were included in this nomogram. The above factors have been shown to be independent prognostic predictors. This nomogram achieved a greater accuracy of CSS than the AJCC-TNM classification. The age at the time of RNU is an independent indicator of patient outcomes 18. The primary recognized prognostic factors are the tumor stage and grade 18-23.

Different nomograms based on the French Collaborative National Database that incorporate several variables have been developed 9-11. In 2012, Eugene K. Cha et al. developed a seven-factor prognostic model for the prediction of CSS after RNU for UTUC 6. The model predicted 26.5% of noninvasive urothelial carcinoma patients. In this study, only age, the T classification, the lymph node status, lymphovascular invasion (LVI), and the tumor architecture were independent predictors of CSS (all *P* values <0.005). However, the grade and concomitant carcinoma *in situ* (CIS) were still incorporated into this model. It could be more helpful to establish a uniform model for daily urological practice than choose among models. In the future, statistical prediction models of this cancer could be evaluated, and those that meet all AJCC criteria should be endorsed.

There are some several limitations as follows: 1) limitations inherent to retrospective analyses; 2) some information was not included in this study; according to previous studies, neoadjuvant chemotherapy and adjuvant chemotherapy significantly benefit overall survival, but the current nomogram was not compared with well-developed models; and 3) we failed to present the difference among established nomograms. However, the clinicopathological features used to establish the nomogram have been used in previous studies. The nomograms did not incorporate the same variables, or have difference prognosis outcomes. Our nomogram only includes 5 of the most common factors. Because some factors could not be collected, such comparisons could not be performed. In addition, we compared the newly developed nomogram with the 8th AJCC TNM classification, which is considered among the most important prediction tools for UTUC. The medical records of the patients included in this study were randomly (7:3) divided into the training and validation cohorts, and number of patients per cohort slightly differed from that in previous studies. Nevertheless, we obtained the same results. Although internal and external validation of the data set was performed, the results still need to be externally verified in large samples. However, we believe that different established nomograms could be verified with unified data.

## Conclusion

A concise novel nomogram that is superior to the current AJCC TNM staging system was established for the prediction of CSS after RNU for invasive UTUC. This model can be an important tool that aids clinical decision making. The clinical value of this tool should be validated in prospective, multi-institutional studies.

## Figures and Tables

**Table 1:** Clinicopathological characteristics of the UTUC patients

Variable	Training set (n=3360)	Validation set (n=1436)	*P*
Age at surgery, year, mean (standard error: SE)	71.6±0.19	72.1±0.27	0.125
**Gender**			0.420
Male	1942 (57.8)	848 (59.1)	
Female	1418 (42.2)	588 (40.9)	
**Race**			0.751
Caucasian	2973 (88.5)	1266 (88.2)	
Other	387 (11.5)	170 (11.8)	
**Tumor location**			0.698
Pelvic tumors	2248 (66.9)	969 (67.5)	
Ureteral and/or multifocal tumors	1112 (33.1)	467 (32.5)	
**T stage**			0.997
T1	1083 (32.2)	458 (31.9)	
T2	663 (19.7)	284 (19.8)	
T3	1403 (41.8)	603 (42.0)	
T4	211 (6.3)	91 (6.3)	
**N stage**			0.297
N0	3053 (90.9)	1312 (91.4)	
N1	182 (5.4)	76 (5.3)	
N2	125 (3.7)	68 (4.7)	
**M stage**			0.179
M0	3263 (97.1)	1384 (96.4)	
M1	97 (2.9)	52 (3.6)	
**Grade**			0.374
G1-2	627 (18.7)	289 (20.1)	
G3-4	2733 (81.3)	1174 (81.8)	
**AJCC-TNM**			0.974
I	1058 (31.5)	446 (31.1)	
II	625 (18.6)	263 (18.3)	
III	1190 (35.4)	514 (35.8)	
IV	487 (14.5)	213 (14.8)	

**Table 2:** Cancer specific mortality in patients with UTUC

Variable	Training set			Validation set		
	3-year CSS	5-year CSS	*P*	3-year CSS	5-year CSS	*P*
Age at surgery (year)			<0.001			0.002
*≤*73	84.5 (82.5-86.5)	78.2 (75.8-80.6)		81.3 (78.2-84.4)	76.1 (72.4-79.8)	
>73	77.2 (74.8-79.6)	68.9 (66.0-71.8)		77.1 (73.4-80.8)	70.1 (65.6-74.6)	
**Gender**			0.002			0.025
Male	83.2 (81.2-85.2)	76.8 (74.4-79.2)		80.5 (77.4-83.6)	75.0 (71.3-78.7)	
Female	78.0 (75.5-80.5)	70.4 (67.5-73.3)		77.2 (73.3-81.1)	70.8 (66.1-75.5)	
**Race**			0.299			0.341
Caucasian	81.4 (79.8-83.0)	74.6 (72.6-76.6)		79.4 (76.9-81.9)	73.7 (70.6-76.8)	
Other	77.5 (72.8-82.2)	70.0 (64.3-75.7)		77.4 (70.1-84.7)	69.9 (60.9-78.9)	
**Tumor location**			0.975			0.693
Pelvic tumors	80.8 (78.8-82.8)	73.7 (71.3-76.1)		79.2 (76.3-82.1)	73.4 (69.9-76.9)	
Ureteral and/or multifocal tumors	81.3 (78.6-84.0)	74.8 (71.5-78.1)		79.1 (74.6-83.6)	72.9 (67.6-78.2)	
**T stage**			<0.001			<0.001
T1	92.3 (90.5-94.1)	88.2 (85.8-90.6)		94.1 (91.7-96.5)	91.1 (87.8-94.4)	
T2	85.9 (82.8-89.0)	79.8 (75.9-83.7)		84.4 (79.3-89.5)	78.8 (72.7-84.9)	
T3	73.8 (71.1-76.5)	64.5 (61.2-67.8)		68.5 (64.0-73.0)	58.3 (52.8-63.8)	
T4	45.2 (36.8-53.6)	30.4 (21.4-39.4)		39.8 (26.1-53.5)	39.8 (26.1-53.5)	
**N stage**			<0.001			<0.001
N0	83.3 (81.5-85.1)	76.6 (74.6-78.6)		81.8 (79.4-84.2)	76.1 (73.2-79.0)	
N1	53.8 (44.6-63.0)	43.3 (33.1-53.5)		50.3 (35.6-65.0)	37.3 (21.8-52.8)	
N2	51.5 (39.5-63.5)	38.1 (21.6-54.6)		38.8 (19.4-58.2)	38.8 (19.4-58.2)	
**M stage**			<0.001			<0.001
M0	82.2 (80.6-83.8)	75.2 (73.2-77.2)		81.0 (78.6-83.4)	74.9 (72.0-77.8)	
M1	24.5 (10.0-39.0)	16.3 (0-32.6)		19.7 (5.0-34.4)	19.7 (5.0-34.4)	
**Grade**			<0.001			<0.001
G1-2	93.5 (91.3-95.7)	90.5 (87.8-93.2)		94.7 (91.8-97.6)	93.5 (90.2-96.8)	
G3-4	77.7 (75.9-79.5)	69.6 (67.4-71.8)		74.5 (71.6-77.4)	66.7 (63.0-70.4)	
**AJCC-TNM**			<0.001			<0.001
I	92.9 (91.1-94.7)	88.7 (86.3-91.1)		94.9 (92.5-97.3)	91.8 (88.7-94.9)	
II	87.2 (84.3-90.1)	81.5 (77.8-85.2)		85.5 (80.6-90.4)	80.3 (74.0-86.6)	
III	76.6 (73.9-79.3)	67.6 (64.1-71.1)		72.6 (67.9-77.3)	62.5 (56.6-68.4)	
IV	50.9 (45.2-56.6)	37.9 (31.2-44.6)		44.5 (35.5-53.5)	38.7 (29.3-48.1)	

**Table 3:** Multivariate analysis of the clinicopathological factors used to predict the cancer specific mortality of patients with UTUC

Variable	Training set			Validation set		
	HR	95% CI	*P*	HR	95% CI	*P*
Age at surgery	1.748	1.50-2.04	<0.001	1.030	1.02-1.04	<0.001
**T stage**			<0.001			<0.001
T1	Ref	Ref	Ref	Ref	Ref	Ref
T2	1.750	1.33-2.30	<0.001	1.580	1.00-2.48	0.049
T3	2.888	2.30-3.52	<0.001	3.670	2.53-5.33	<0.001
T4	5.753	4.27-7.75	<0.001	6.720	4.05-11.16	<0.001
**N stage**			<0.001			0.001
N0	Ref	Ref	Ref	Ref	Ref	Ref
N1	1.511	1.16-2.00	0.003	1.830	1.23-2.72	0.003
N2	1.696	1.23-2.34	0.001	2.030	1.23-3.36	0.006
**M stage**						<0.001
M0 vs. M1	4.229	3.05-5.86	<0.001	3.371	2.20-5.17	
**Grade**						<0.001
G1-2 vs. G3-4	1.207	1.50-2.04	0.001	1.400	1.18-1.66	

**Table 4:** Predictive accuracy of the staging system

Stage	C-index (Training set)	Bootstrap C-index (Training set)	C-index (Validation set)
AJCC-TNM	0.71	0.70	0.73
Nomogram	0.74	0.75	0.77

## Abbreviations

AJCC/UICC: American Joint Committee on Cancer/Union Internationale Contre le Cancer
AUC: receiver-operating characteristic curve
CSS: cancer-specific survival
EAU: European Association of Urology
NCCN: National Comprehensive Cancer Network
RNU: radical nephroureterectomy
SEER: Surveillance, Epidemiology and End Results
TNM: tumor-node-metastasis
UC: urothelial carcinomas
UTUC: upper tract urothelial carcinoma
